# Topical Neck Cooling Without Systemic Hypothermia Attenuates Myocardial Ischemic Injury and Post-ischemic Reperfusion Injury

**DOI:** 10.3389/fcvm.2022.893837

**Published:** 2022-06-28

**Authors:** Aimee Zhang, Radhika Rastogi, Katherine M. Marsh, Boris Yang, Di Wu, Irving L. Kron, Zequan Yang

**Affiliations:** Department of Surgery, University of Virginia Health System, Charlottesville, VA, United States

**Keywords:** therapeutic hypothermia, myocardial infarction, topical hypothermia, ischemia-reperfusion injury, vagal activation

## Abstract

**Background:**

Following acute myocardial infarction (MI), irreversible damage to the myocardium can only be reduced by shortening the duration between symptom onset and revascularization. While systemic hypothermia has shown promising results in slowing pre-revascularization myocardial damage, it is resource intensive and not conducive to prehospital initiation. We hypothesized that topical neck cooling (NC), an easily implemented therapy for en route transfer to definitive therapy, could similarly attenuate myocardial ischemia-reperfusion injury (IRI).

**Methods:**

Using an *in vivo* mouse model of myocardial IRI, moderate systemic hypothermia or NC was applied following left coronary artery (LCA) occlusion and subsequent reperfusion, at early, late, and post-reperfusion intervals. Vagotomy was performed after late NC in an additional group. Hearts were harvested to measure infarct size.

**Results:**

Both hypothermia treatments equally attenuated myocardial infarct size by 60% compared to control. The infarct-sparing effect of NC was temperature-dependent and timing-dependent. Vagotomy at the gastroesophageal junction abolished the infarct-sparing effect of late NC. Cardiac perfusate isolated following ischemia had significantly reduced cardiac troponin T, HMGB1, cell-free DNA, and interferon α and β levels after NC.

**Conclusions:**

Topical neck cooling attenuates myocardial IRI in a vagus nerve-dependent manner, with an effect comparable to that of systemic hypothermia. NC attenuated infarct size when applied during ischemia, with earlier initiation resulting in superior infarct sparing. This novel therapy exerts a cardioprotective effect without requiring significant change in core temperature and may be a promising practical strategy to attenuate myocardial damage while patients await definitive revascularization.

## Introduction

Ischemic heart disease remains the single leading cause of death in the United States, with acute myocardial infarction (MI) accounting for the considerable morbidity and mortality from this condition (1). Final infarct size, which is the main predictor of outcomes following MI, is directly related to the duration of time from symptom onset to definitive treatment restoring coronary flow and myocardial tissue perfusion (2–5). Thus, the key to maximal salvage is summarized by the phrase “Time is Muscle”—shortening patients' symptom-to-door and door-to-balloon time in order to minimize the final volume of infarcted myocardium (4, 6, 7). Strategies to reduce the final infarct size include implementation of mild to moderate systemic hypothermia, which has been shown to protect against MI and slow the rate of infarction, allowing for increased ischemic time prior to reperfusion without increasing infarct size (8, 9). Multiple clinical trials have since evaluated the effect of endovascular cooling during primary percutaneous coronary intervention (PCI). It has been shown that patients who are systemically cooled to <35°C early during MI have a significant reduction in infarct size at the time of reperfusion (10). However, the induction of systemic hypothermia is resource intensive and must be performed with supervision by medical professionals, making the challenge of initiating these therapies rapidly after initial symptom onset likely unsurmountable (7).

We recently identified an alternative therapeutic hypothermia approach, topical neck cooling (NC), which could potentially exert similar infarct-sparing effects to systemic hypothermia *via* stimulation of the vagus nerve by temperature reduction. The link between the vagus nerve and cardioprotection has been reported by our lab (11) and others (12–16), and the vagus nerve has been demonstrated to be directly activated by temperature changes (17). In the current study, we investigated the cardioprotective effect of topical neck cooling, hypothesizing that locally decreased temperatures in the soft tissue and encompassed structures of the neck, without inducing systemic hypothermia, would attenuate myocardial ischemic and reperfusion injury (IRI). Furthermore, we evaluated the role of the timing of NC application and the depth of NC in achieving a protective effect against myocardial IRI. This topical approach to hypothermic therapy, which is non-invasive and easily implemented after MI, may prolong the therapeutic window to reach definitive reperfusion interventions and thereby could lead to improved patient outcomes.

## Methods

This study complied with the Guide for the Care and Use of Laboratory Animals as recommended by the U.S. National Institutes of Health ensuring that all animals received humane care. The University of Virginia Animal Care and Use Committee reviewed and approved the study protocol.

### Animals and Experimental Protocols

**Figure 1:**
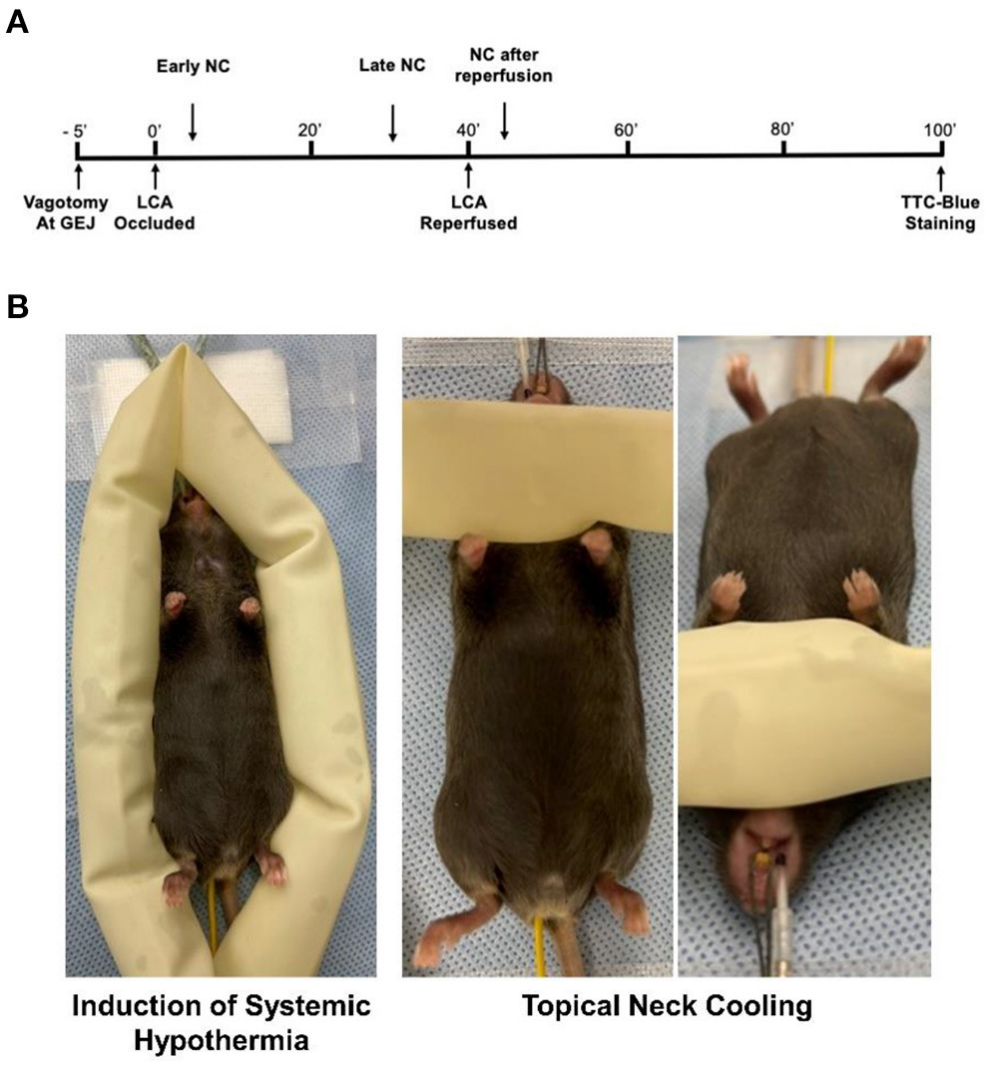
Experimental protocol. **(A)** C57BL6 mice underwent 40 min of LCA occlusion followed by 60 min of reperfusion before infarct size was evaluated by TTC-Phthalo blue staining. Topical neck cooling experimental groups underwent early cooling starting 5 min after LCA occlusion to 5 min after reperfusion, late cooling from 30 min after LCA occlusion to 30 min after reperfusion, late cooling after vagotomy at gastroesophageal junction (GEJ) performed 5 min prior to LCA occlusion, or cooling from 5 min after reperfusion to 45 min after reperfusion. **(B)** Methods to achieve moderate systemic hypothermia and topical neck cooling. NC, topical neck cooling; GEJ, gastro-esophageal junction; LCA, left coronary artery;' min.

C57BL/6 wild type mice (male and female aged 9–12 weeks, purchased from The Jackson Laboratory, Bar Harbor, ME) were used in the study. Mice underwent 40 min of left coronary artery (LCA) occlusion (ischemia) followed by 60 min of reperfusion (40′/60′ IRI). Systemic hypothermia or NC was applied 5 min after LCA occlusion for 40 min. Late NC was started 10 min before reperfusion. In an additional group, NC was started 5 min after reperfusion for 40 min. Vagotomy at the gastroesophageal junction (GEJ), when performed, was completed 5 min prior to LCA occlusion. Myocardial infarct size was evaluated both at the completion of the ischemic period and at the completion of the reperfusion period. Cardiac perfusate (CP) was collected at the end of 40 min of ischemia (Figure 1A).

### Surgical Procedures to Induce Myocardial Infarction in Intact Mice

The details of the surgical procedure with a video are published in JTCVS (18). Briefly, mice were anesthetized with intraperitoneal Avertin (Tribromoethanol) at 250 mg/kg, placed in supine position, and orally intubated with a PE-60 tube. Respiration was maintained with a rodent ventilator with room air, at a frequency of 130 strokes/min and a tidal volume of 8–10 μl/g weight. The left pleural cavity was entered by cutting the intercostal muscles and left 3rd and 4th ribs with a cautery pen and scissors to expose the heart. An 8-0 Prolene suture was passed beneath the LCA 1 mm inferior to the left atrium and tied over a short piece of PE-60 tubing to occlude the LCA for 40 min. Significant ECG changes, including widening of the QRS and elevation of the ST segment complex (monitored with a PowerLab data recording unit, ADInstruments), and color changes in the risk region were used to confirm successful LCA occlusion. Reperfusion was achieved by untying the ligature and removing the PE-60 tubing. Anesthesia was maintained with additional Avertin doses of 125 mg/kg given every 30 min. Core body temperature was monitored throughout the experiment with a rectal thermocouple interfaced to a digital thermometer (Omega Co).

### Determination of Myocardial Infarct Size

After 40 min of ischemia with or without 60 min of reperfusion, mice were euthanized under deep anesthesia and the heart was excised, cannulated through the ascending aorta with a blunted 23-gauge needle, and perfused with 3 ml 37°C 1% triphenyltetrazolium chloride (TTC) in phosphate buffered saline (PBS, pH = 7.4). The LCA was then re-occluded by retying the suture left around LCA. The heart was then perfused with 0.3–0.5 ml 2% Phthalo Blue (Heubach Ltd, Fairless Hills, PA) to delineate the non-ischemic tissue. The left ventricle was cut into 5–7 transverse slices and fixed in 10% neutral buffered formalin solution. Each slice was weighed and photographed. The sizes of the non-ischemic area, the risk region, and the infarct area were calculated as a percentage of the corresponding slice multiplied by weight of the slice (18–21).

### Vagotomy at the Gastroesophageal Junction

After induction of general anesthesia and intubation, a vertical midline epigastric incision was sharply made. The stomach was retracted caudally to expose the GEJ. The anterior and posterior vagal nerve trunks were isolated and divided. The laparotomy was closed in layers using 5-0 Nylon.

### Systemic Hypothermia and Topical Neck Cooling

Systemic hypothermia was achieved by encircling the mice with ice-filled 3/4-inch rubber tubing to achieve a core rectal temperature between 30 and 32°C. NC was achieved by wrapping the ventral neck with ice-filled 3/4-inch rubber tubing (Figure 1B). To achieve a milder depth of localized cooling, an additional group underwent mild NC with application of rubber tubing filled with 20°C water. The cooling appliances were exchanged at frequent intervals to maintain a consistent temperature until the full treatment period was completed. In all NC groups, rectal temperature was maintained between 36.0 and 37.0°C using a heating lamp.

### Analysis of Cardiac Perfusate

Following 40 min of LCA occlusion only, the hearts were harvested, cannulated through the ascending aorta with a blunted 23-gauge needle, and perfused with 500 μl of 37°C PBS (pH = 7.4) cycled three times. The CP was collected and centrifuged at 3,000 rpm for 20 min and then recollected after cellular sediments were discarded. Levels of cell-free DNA (cfDNA) were evaluated using Nanodrop; levels of high mobility group box 1 (HMGB1), cardiac troponin T (cTnT), and interferon alpha (IFNα) and beta (IFNβ) were evaluated using Western Blot (2, 18). Antibodies to HMGB1, cTnT, IFNα, and IFNβ were purchased from Abcam and ThermoFisher respectively.

### Statistical Analysis

Comparisons between groups were performed with one-way analysis of variance with Bonferroni's correction for multiple comparisons and unpaired Student's *t*-test. Paired Student's *t*-test was used to analyze changes in heart rate. Prism 7 (GraphPad Software Inc., La Jolla, CA) was used to perform statistical calculations. Data are presented as mean±standard error of the mean, with a *p*-value of 0.05 indicating statistical significance. Drs. Katherine Marsh and Zequan Yang had full access to all data in the study and take responsibility for its integrity and data analysis.

## Results

### Systemic Hypothermia and Topical Neck Cooling Equally Attenuate Myocardial Ischemia/Reperfusion Injury (IRI)

**Figure 2:**
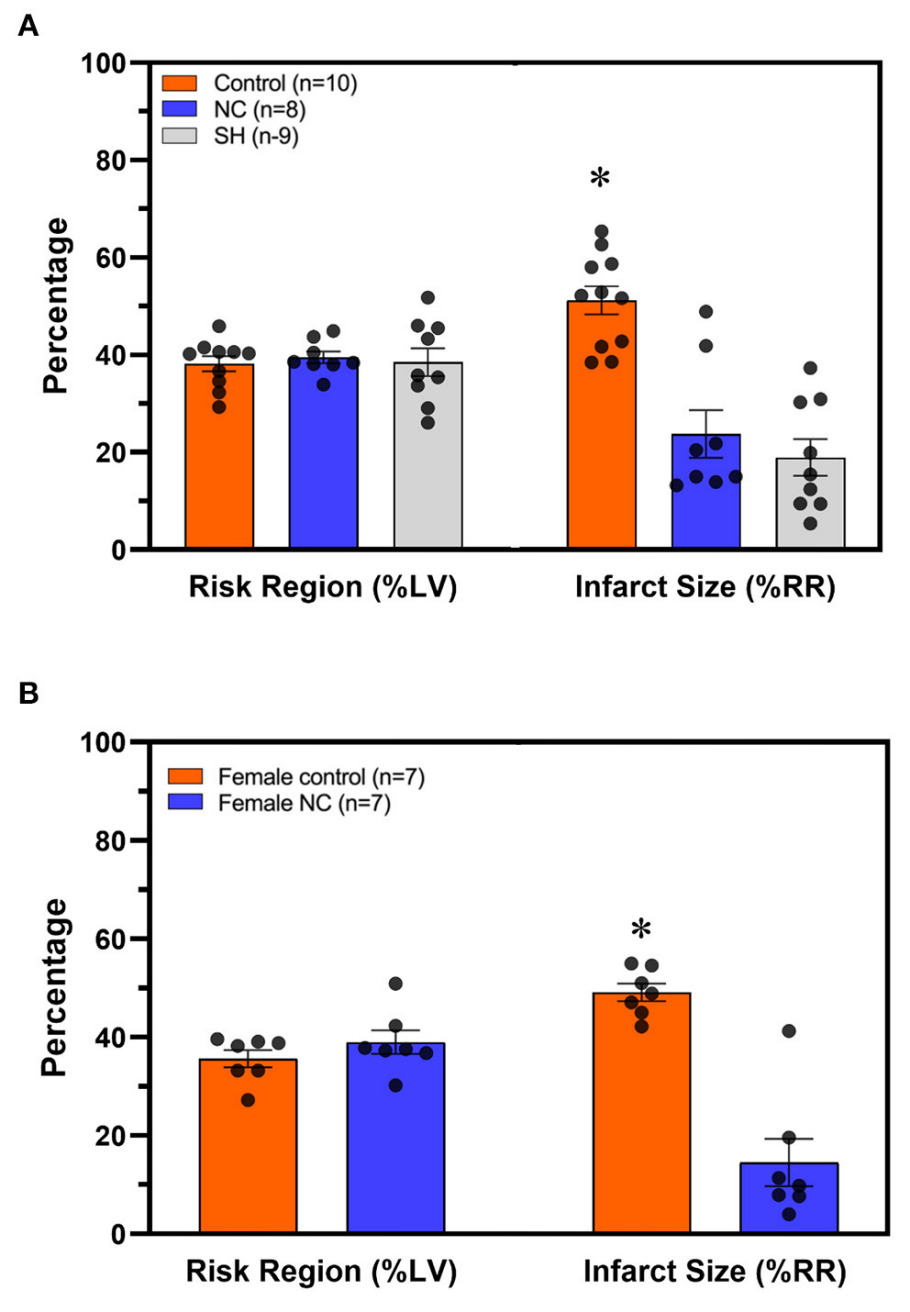
Topical neck cooling exerts a comparable infarct-sparing effect to systemic hypothermia. **(A)** Risk region and infarct size were calculated in male mice that underwent systemic hypothermia or topical neck cooling and compared to control mice following myocardial ischemia-reperfusion injury. **(B)** Similar results of infarct-sparing with topical neck cooling compared to control were observed in female mice as compared to male mice following myocardial ischemia-reperfusion injury. RR, risk region; LV, left ventricle; NC, topical neck cooling; SH, systemic hypothermia; * *p* < 0.05 vs. other groups.

C57BL6 male mice underwent 40 min of LCA occlusion and 60 min of reperfusion (40′/60′ IRI). Five minutes after LCA occlusion, mice underwent either systemic hypothermia to achieve a core body temperature of 30–32°C or NC to achieve a neck subcutaneous tissue temperature of 14–17°C for 40 min. Risk regions (ischemic area as a percentage of left ventricle, LV, mass) were comparable among the control and hypothermic groups (*p* = NS, Figure 2A). Myocardial infarct size (IS, as a percentage of the risk region area) was 53 ± 3% in normothermic control mice. Systemic hypothermia during ischemia reduced IS to 19 ± 4% (*p* < 0.05 vs. control). NC reduced IS to 24 ± 5% (*p* < 0.05 vs. control, *p* = NS vs. systemic hypothermia, Figure 2A). In female C57BL6 mice undergoing 40′/60′ IRI, risk regions were also comparable (36 ± 2% NC vs. 39 ± 2% control, *p* = NS) between the normothermic control and NC groups, and NC similarly attenuated IS (15 ± 5% NC vs. 49 ± 2%, *p* < 0.05, Figure 2B).

### The Infarct-Sparing Effect of Neck Cooling Is Temperature-Dependent and Requires Early Application During Ischemia

**Figure 3:**
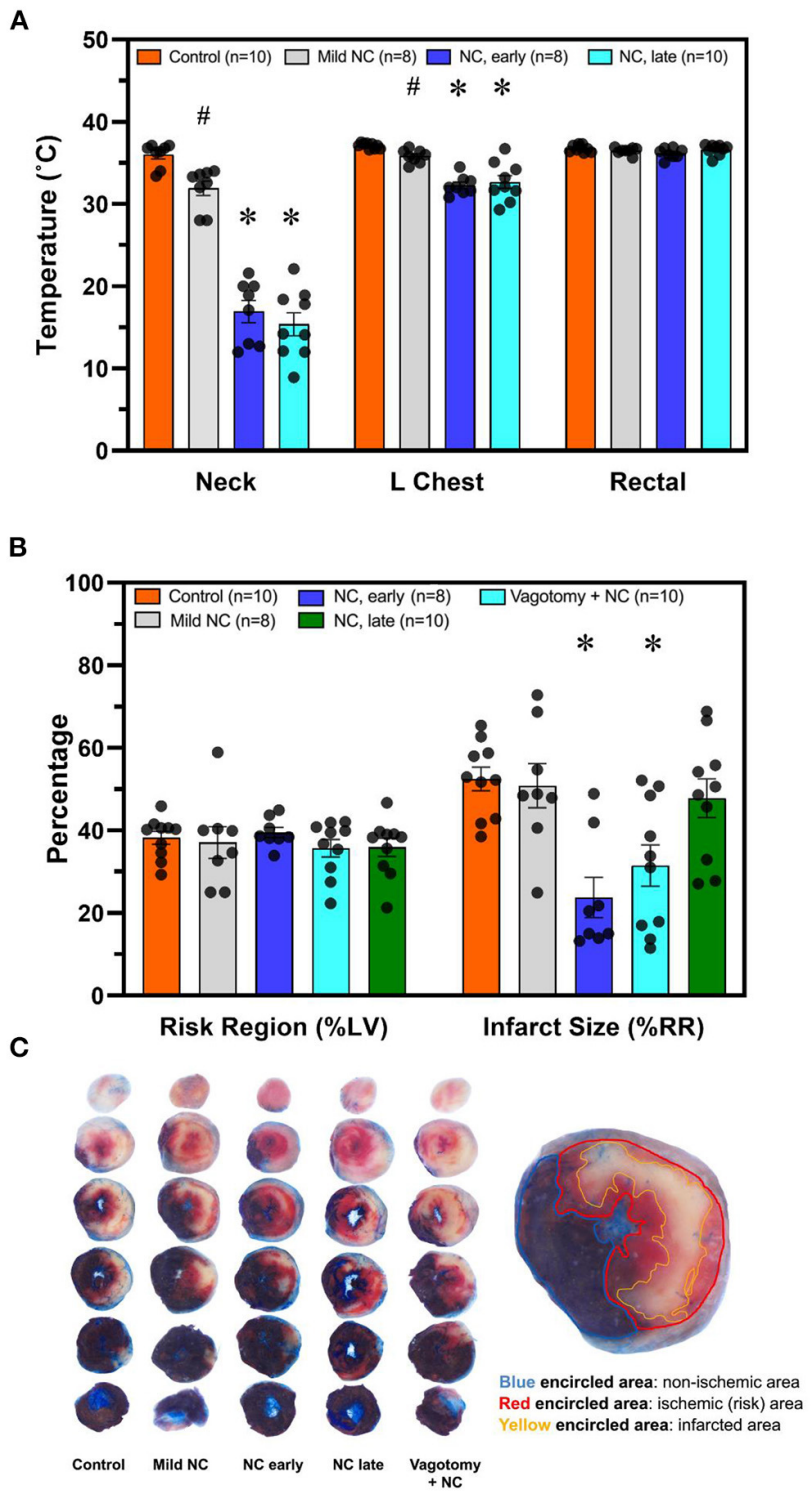
NC without systemic hypothermia attenuates myocardial infarct size in a vagus nerve- and temperature-dependent manner, with earlier initiation resulting in superior infarct sparing effect. **(A)** Temperature was measured 10 min after initiation of topical neck cooling interventions in the left pleural place and neck subcutaneous tissue. Core temperature was measured rectally. **(B)** Myocardial infarct size was calculated as a percentage of left ventricle area at risk after 40 min of left coronary artery occlusion followed by 60 min of reperfusion. Application of topical neck cooling interventions, as well as the effect of vagotomy at the gastroesophageal junction, was compared to normothermic control. **(C)**. Representative TTC-Blue staining left ventricle slices to determine RR and IS. *Mild NC* topical neck cooling with tubing containing room-temperature water; *early NC* topical neck cooling with ice-filled tubing initiated 5 min after LCA occlusion*; late NC* topical neck cooling with ice-filled tubing initiated 10 min prior to reperfusion*; vagotomy* + *NC* vagotomy at the gastro-esophageal junction prior to left coronary occlusion and initiation of topical neck cooling with ice-filled rubber-tubing 10 min prior to reperfusion. LCA, left coronary artery; RR, risk region; LV, left ventricle; IS, infarct size. * *p* < 0.05 vs. control and mild NC; # *p* < 0.05 vs. control only.

**Figure 4:**
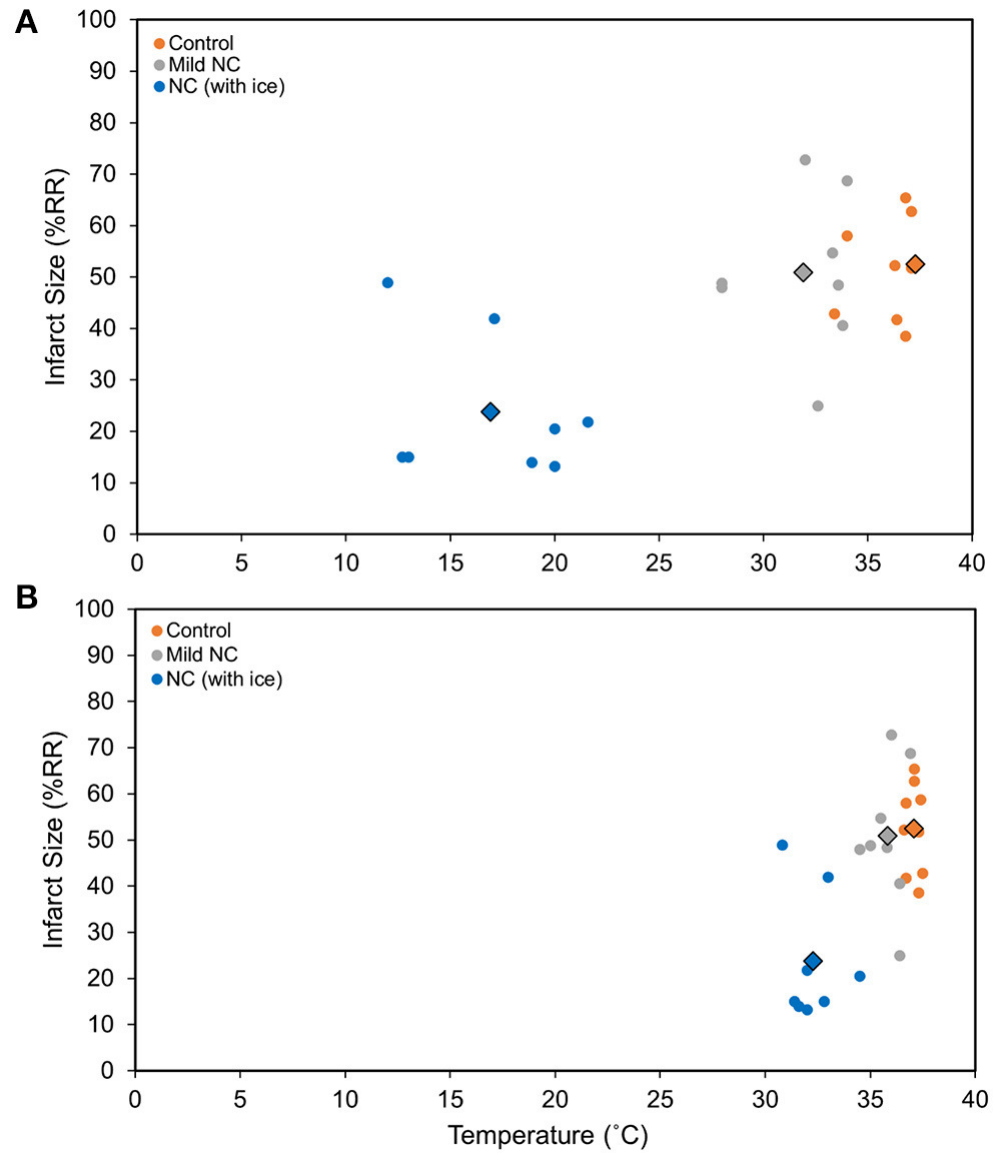
NC attenuates myocardial infarct size in a vagus nerve- and temperature-dependent manner. **(A)** Neck temperature during implemented therapy compared to infarct size. **(B)**. Chest temperature during implemented therapy compared to infarct size. Myocardial infarct size was calculated as a percentage of left ventricle area at risk after 40 min of left coronary artery occlusion followed by 60 min of reperfusion. The average temperature vs. average infarct size for each group is demonstrated with the outlined diamond. *Mild NC* topical neck cooling with tubing containing room-temperature water; *NC (with ice)* topical neck cooling with ice-filled tubing initiated 5 min after LCA occlusion. RR, risk region.

C57BL6 male mice underwent 40′/60′ IRI. NC was achieved with either room-temperature (20°C) water-filled 3/4-inch rubber tubing to achieve mild NC or ice-filled 3/4-inch rubber tubing applied 5 min after LCA occlusion for a 40-min duration. In two additional groups, NC was initiated later, starting 10 min prior to reperfusion, with ice-filled tubing with or without vagotomy at the level of GEJ. Temperatures were measured 10 min after NC interventions in all groups in the neck subcutaneous tissue, in the left pleural space, and rectally. In the normothermic control, mild NC, early NC, and late NC groups, neck subcutaneous temperatures were 37.3, 31.9, 16.9, and 15.4°C respectively, left pleural cavity temperatures were 37.1, 35.8, 32.3, and 32.7°C, and rectal temperatures were 36.8, 36.5, 36.2, and 36.6°C (Figure 3A). Mild NC mice had significantly lower neck and left pleural temperatures than normothermic controls (*p* < 0.05, Figure 3A), but IS was comparable between these groups (*p* = NS, Figures 3B, 4). Both NC (early and late) groups had significantly lower neck and left pleural temperatures and smaller IS than the mild NC and normothermic control groups (*p* < 0.05, Figures 3A,B, 4). However, the late NC group had a significantly larger IS than the group with early initiation of NC. The infarct-sparing effect of NC was eliminated by vagotomy at the GEJ (Figure 3B). The infarct-sparing effect of NC also disappeared when application was initiated after the onset of reperfusion (NC for 40 min starting 5 min after reperfusion, IS 53 ± 3%, *p* = NS vs. control).

### Neck Cooling Attenuates Ischemic Myocardial Injury

**Figure 5:**
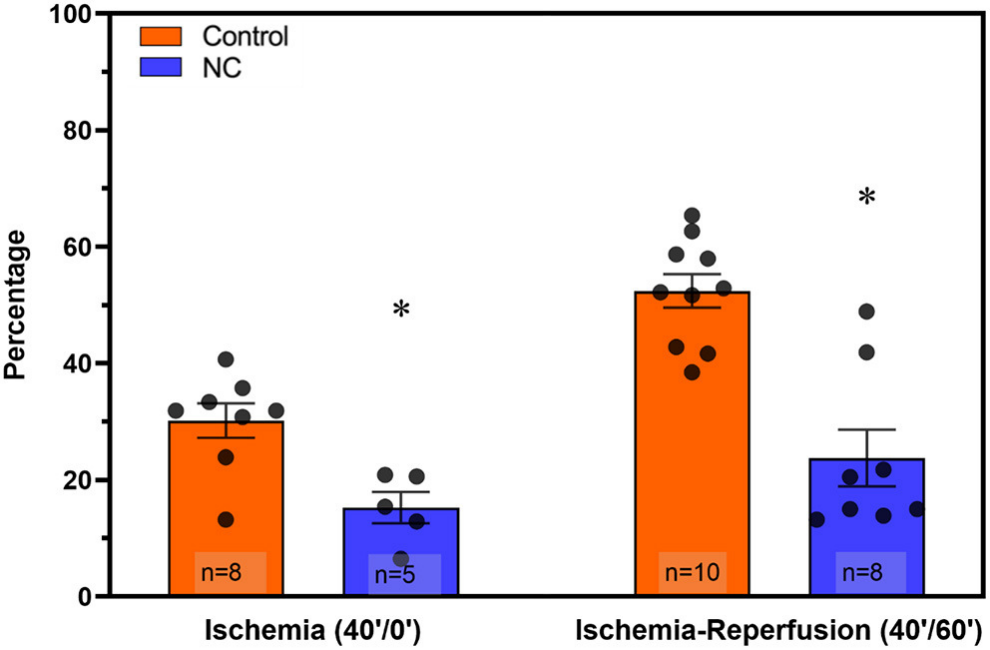
NC attenuated both ischemic myocardial injury and post-ischemic reperfusion injury. Hearts were harvested from normothermic control and NC-treated mice at the end of 40 min of ischemia (40′/0′, left) and at the end of 40 min of ischemia followed by 60 min of reperfusion (40′/60′, right) to evaluate infarct size by TTC-blue staining (calculated as a percentage area of the left ventricle region at risk). NC, topical neck cooling; * *p* < 0.05 vs. corresponding control.

**Figure 6:**
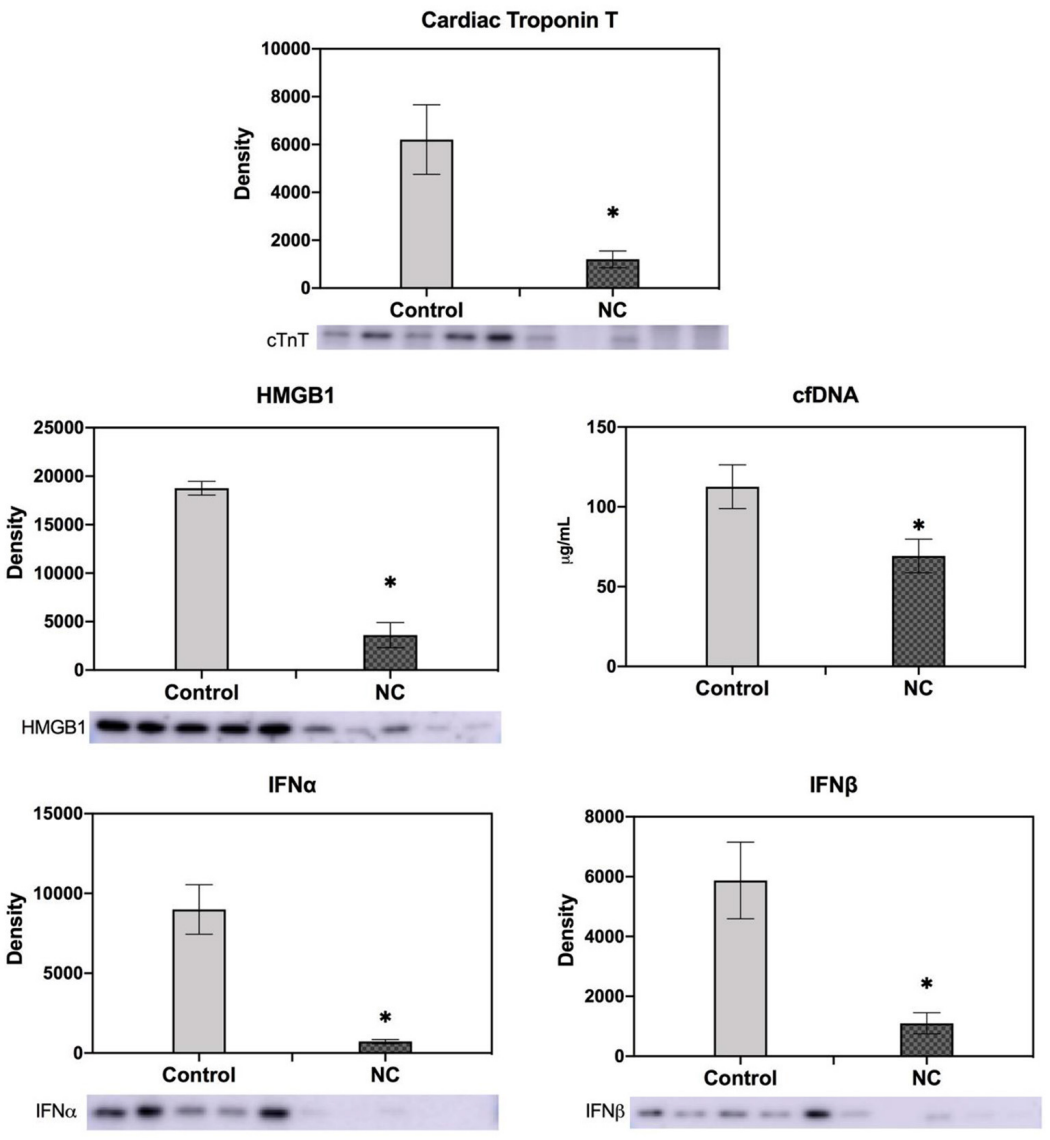
NC decreased cardiac troponin T, HMGB1, cell-free DNA, and type I interferon (IFNα and IFNβ) levels in cardiac perfusate after ischemia. Cardiac perfusate was collected after 40 min of left coronary artery occlusion without reperfusion from normothermic mice or mice that underwent NC. Levels of cfDNA were measured by Nanodrop; levels of cTnT, HMGB1, and IFNα and IFNβ were measured by Western Blot. NC, topical neck cooling; cTnT, cardiac troponin T; cfDNA, cell-free DNA; * *p* < 0.05 vs. control.

**Figure 7:**
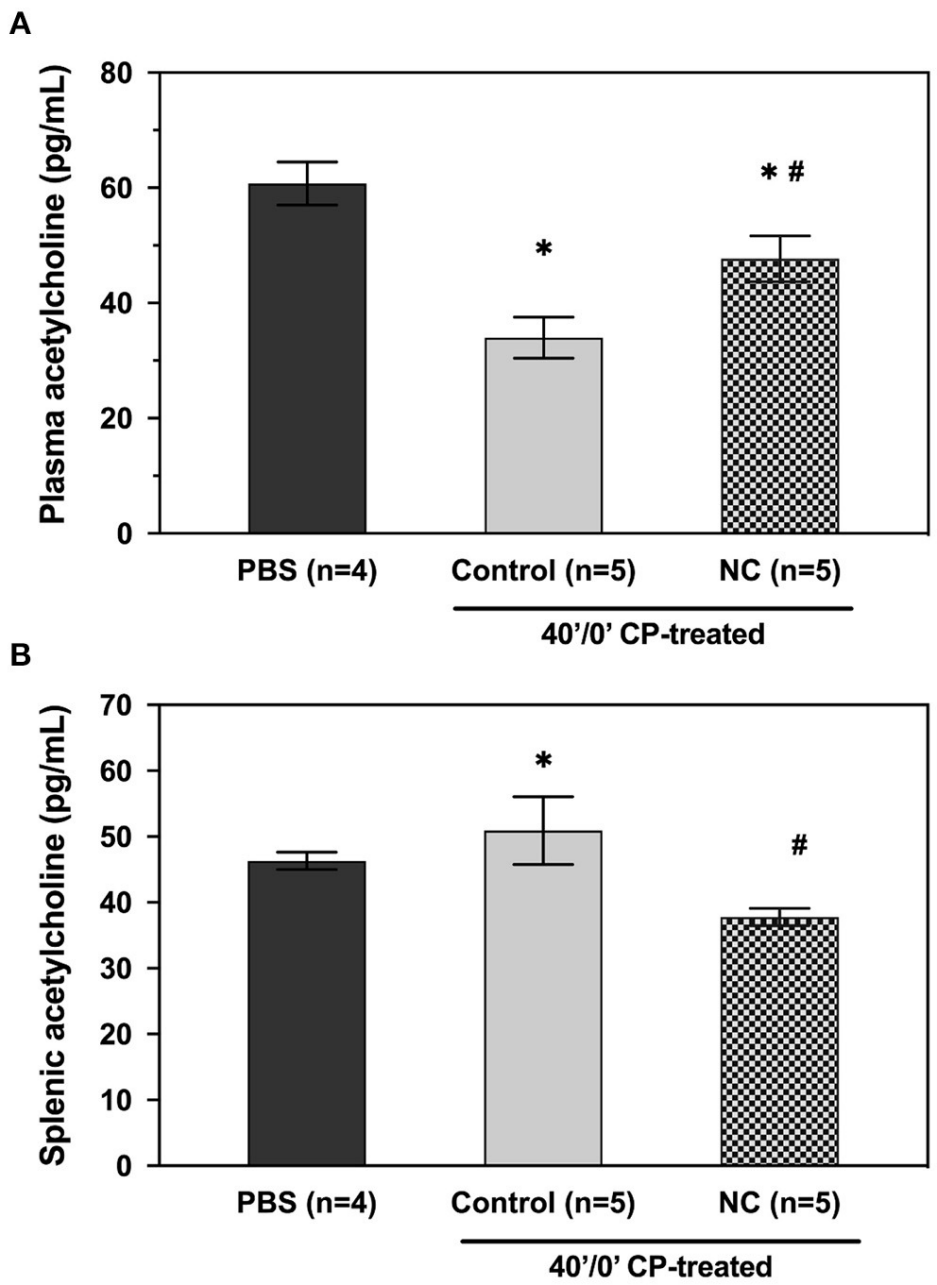
NC attenuates the effect of post-ischemia cardiac perfusate on plasma **(A)** and splenic **(B)** acetylcholine levels. Cardiac perfusate was isolated following 40 min of ischemia and administered to naive mice at a dose of 2 μl/g i.v. bolus. Plasma and splenic acetylcholine levels were then evaluated in treated groups that remained normothermic or underwent NC. PBS, phosphate-buffered saline; NC, topical neck cooling; CP, cardiac perfusate;' min; * *p* < 0.05 vs. PBS-treated group; # *p* < 0.05 vs. CP-treated control group.

Mice underwent 40 min of LCA occlusion without reperfusion (40′/0′ IRI) to evaluate ischemia-induced injury. NC was applied 5 min after LCA occlusion for 35 min. After 40 min of ischemia, hearts were harvested to collect CP and perform TTC-Phthalo Blue staining to calculate IS. IS in normothermic mice was 30 ± 3% compared to 15 ± 3% in NC mice (*p* < 0.05, Figure 5). The 40′/0′ IS was 60% of the IS after 40′/60′ IRI with or without corresponding neck cooling (*p* < 0.05, Figures 2A, 4). NC also significantly reduced cTnT, HMGB1, cfDNA, IFNα, and IFNβ in CP (*p* < 0.05 vs. control, Figure 6). Next, CP from control mice with 40′/0′ IRI was administered to naive mice without IRI at a treatment dose of 2 μl/g i.v. bolus. An additional group of naïve mice was treated with PBS at the same volume with a 2 μl/g i.v. bolus. In normothermic mice 15 min after treatment, 40′/0′ CP decreased plasma acetylcholine levels and increased splenic tissue acetylcholine levels compared to PBS administration (*p* < 0.05, Figure 7). NC attenuated this decrease in plasma acetylcholine following CP administration (*p* < 0.05 vs. PBS & vs. CP control) and abolished the change in splenic tissue acetylcholine levels (*p* = NS vs. PBS, *p* < 0.05 vs. CP control).

### Neck Cooling Without Systemic Hypothermia Slows Heart Rate

**Figure 8:**
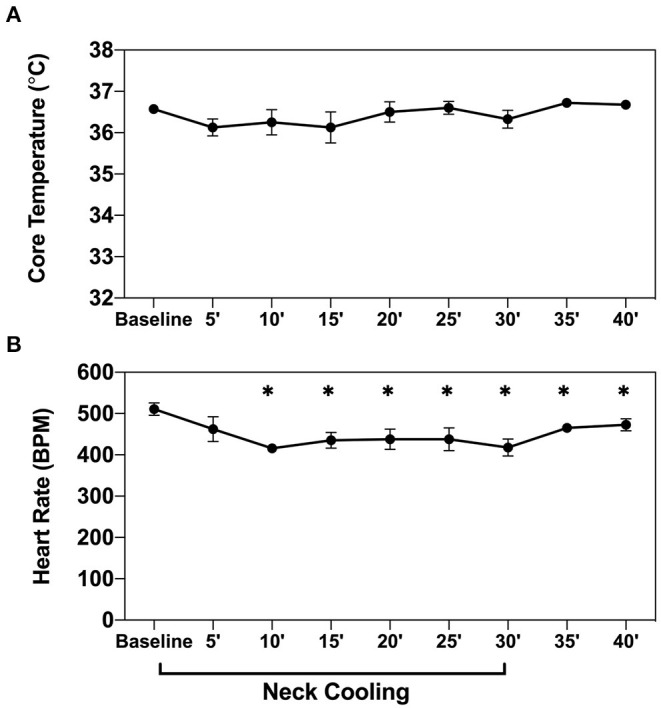
NC slows heart rate without inducing systemic hypothermia. Core body temperature **(A)** and heart rate **(B)** were measured in naive wild-type mice (*n* = 4) before and then every 5 min after application of topical neck cooling for 30 min, followed by a 10-min recovery period. *NC* topical neck cooling;' min; * *p* < 0.05 vs. baseline measurement.

Baseline heart rate measured in naive anesthetized mice (*n* = 4) was 511 ± 15 beats per minute (bpm). After 10 min of NC treatment, the mean heart rate decreased to 416 ± 10 bpm, an 18% reduction from baseline (*p* < 0.05). This significant heart rate reduction was sustained throughout the remainder of the neck cooling period (30 min) and persisted after removal of the cooling appliance. This effect was observed without any corresponding significant change in core body temperature (Figure 8).

## Discussion

Using a mouse model of myocardial IRI, we found that NC produced an infarct-sparing effect comparable to that seen with moderate systemic hypothermia. We distinguished that the cardioprotective effect of NC is timing- and dose-dependent; this therapy optimally should be applied early after initial symptom onset and prior to reperfusion. Furthermore, we identified that NC exerted a cardioprotective effect against both ischemic injury and post-ischemic reperfusion injury. The mechanism of this intervention involves activation of the vagal acetylcholine anti-inflammatory pathway to increase the myocardium's resistance to ischemia and reduce inflammatory responses during post-ischemic reperfusion.

Following the onset of an MI, the key to salvaging ischemic myocardium is shortening the time between coronary artery occlusion and reperfusion of the ischemic myocardium (2–5). Early recognition of MI and timely transportation to the hospital remain the only opportunities to shorten ischemic duration (4, 22), but faster transportation to interventional centers is improbable in the current healthcare landscape, and thus far, clinical treatments to protect myocardium during the ischemic period are lacking (7, 23). At this time, most potential therapies target early reperfusion as an adjunct to definitive revascularization (23). During the ischemic period, coronary occlusion precludes delivery of medication to the at-risk myocardium, rendering pharmaceutical interventions ineffectual during the ischemia phase of acute MI. Moreover, several promising preclinical drug therapies have not shown consistent benefit in clinical trials (7, 24). Pre-conditioning, by either ischemic or pharmacologic methods, prior to MI may increase myocardial tolerance to ischemic insult and attenuate ischemic injury (23, 25–28). However, its clinical application in acute MI is not feasible and often can only be delivered in the peri-revascularization period. Furthermore, randomized controlled trials of remote ischemic preconditioning under controlled elective situations have failed to show consistent clinically relevant beneficial effects (27, 29). When used during the MI itself, smaller clinical trials have suggested benefit with remote ischemic conditioning but a large phase III trial showed no difference in heart failure and death after a year (30–32). Mild to moderate systemic hypothermia has been demonstrated to be protective against acute MI and allows for prolonged time to reperfusion without a concomitant increase in infarct size (8–10, 31). This therapeutic approach also allows for its cardioprotective effect to be implemented during both the ischemic and reperfusion phases of acute MI. However, induction of systemic hypothermia is resource intensive and cannot be performed without the presence of medical personnel and thus has been difficult to translate clinically (7, 31). Thus, this practice has not been implemented as part of pre-hospital management of acute coronary syndrome, and a large gap in patient care persists.

To circumvent the logistical difficulties of inducing systemic hypothermia, we developed a therapeutic hypothermia approach of topical neck cooling and demonstrated that it attenuates myocardial IRI to a similar degree to systemic hypothermia (Figure 2). It has been reported that mild (35°C) and, to an even more significant degree, moderate (32°C) systemic hypothermia result in an infarct-sparing effect (33). Multiple clinical trials have since evaluated the effect of endovascular cooling during primary PCI, and it has been shown that patients with anterior STEMI who were cooled to <35°C early during MI have a significant reduction in infarct size at the time of reperfusion (10). Using an *in vivo* mouse model undergoing 40 min of LCA occlusion and 60 min of reperfusion, we found that moderate systemic hypothermia attenuates myocardial infarct size by 60% in comparison to normothermic mice. By cooling the neck to 15°C while maintaining normal core temperature, we found that simple topical neck cooling produced a similar infarct-sparing effect to that seen with moderate systemic hypothermia (Figure 2A). The infarct-sparing effect of NC is dose-dependent and effective only when it is applied before reperfusion (Figure 3). Given that the neck subcutaneous tissue temperature is similar between mild NC, which did not result in an infarct-sparing effect, and systemic hypothermia (data not shown), the cardioprotective mechanisms underlying NC may differ from that of systemic hypothermia.

**Figure 9:**
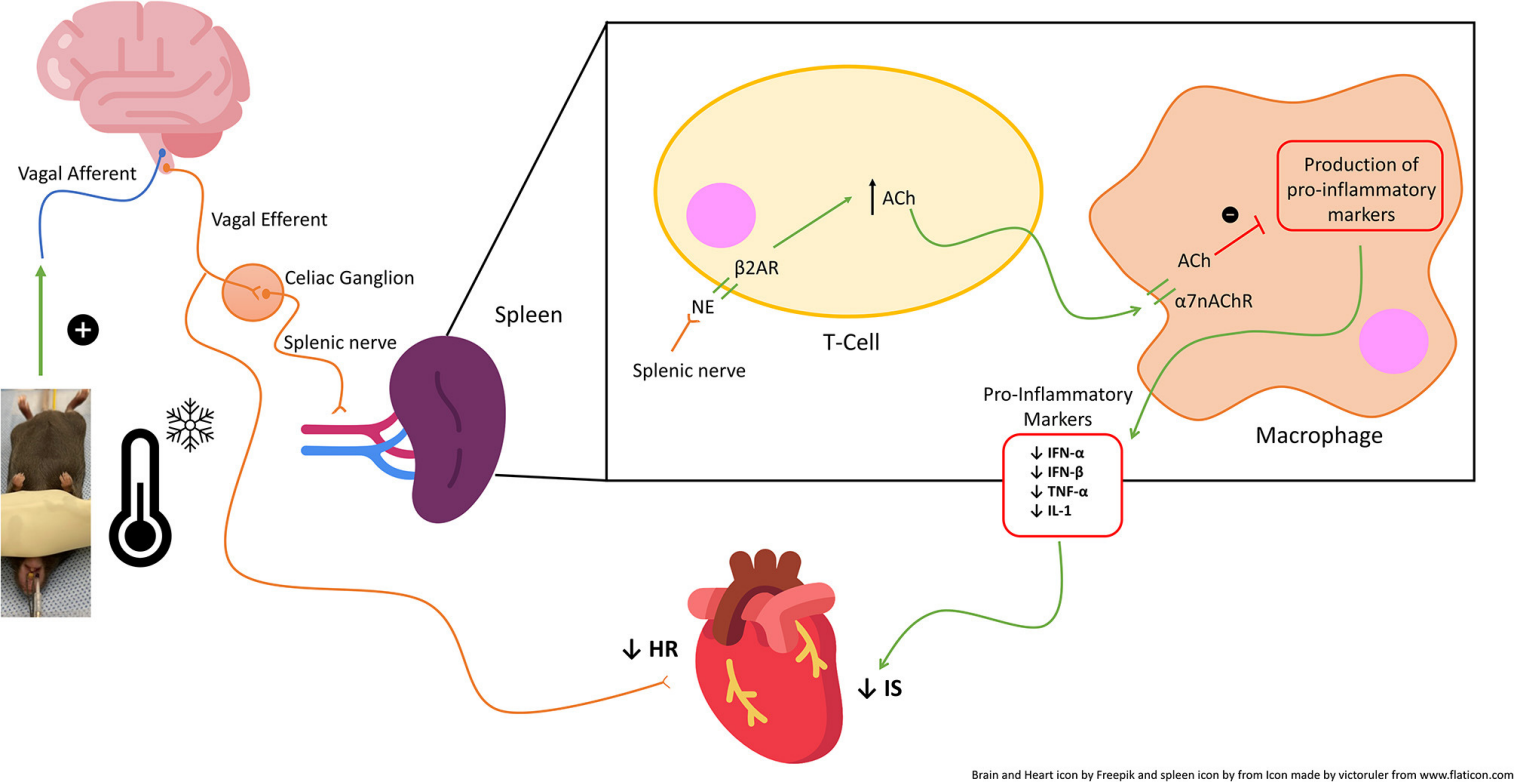
Schematic of the proposed vagal-mediated cholinergic anti-inflammatory pathway (CAP) and mechanism of action of topical neck cooling. Neck cooling activates the vagal nerve which acts *via* the spleen and peripheral immune system to increase acetylcholine and thus inhibit macrophage production of splenic and peripheral pro-inflammatory markers, thus reducing infarct size (IS). NE, norepinephrine; Ach, Acetylcholine.

Therapeutic hypothermia produces multifactorial beneficial effects resulting in an overall protective anti-inflammatory state, inhibition of apoptosis, and activation of cell survival pathways (34, 35). One of the mechanisms underlying hypothermic cardiac protection is vagal activation to modulate the IRI-associated inflammatory response (17, 36), possibly *via* a postsynaptic process (36). Preclinical and small clinical studies have demonstrated electrical vagal nerve stimulation itself has been clinically effective in reducing infarct size (14–16, 31). Moreover, neck cooling (*via* the application of a neck wrap containing frozen ice packs) (37) and electrical stimulation of the vagus nerve at the neck have been shown to relieve migraine headaches (38), raising the possibility that activation of the vagus nerve could be a shared mechanism underlying the beneficial effects of neck cooling In support of this, a recent clinical trial demonstrated that topical neck cooling at the lateral neck stimulates the vagus nerve and produces a parasympathetic nervous system response in healthy participants (39). In the current study, the infarct-sparing effect of NC was abolished by vagotomy at the GEJ (Figures 3B,C), further suggesting that the cardioprotective effect of NC against IRI is mediated by vagus nerve stimulation in the neck region. There was also evidence of parasympathetic activation by NC, with a significant decrease in heart rate observed during NC (Figure 8). In addition to being a major component of the parasympathetic nervous system, the vagus nerve has several other cardioprotective mechanisms (40), and has a recognized anti-inflammatory role with its efferent fibers mediating the cholinergic anti-inflammatory pathway (CAP) (41). The CAP is mediated through the binding of acetylcholine released from distal vagus nerve terminals to α-7-nicotinic cholinergic receptors (α7nAChR) to ultimately inhibit the release of pro-inflammatory cytokines, such as TNFα, by peripheral macrophages (42) and from the spleen (Figure 9) (43). In this study, NC significantly decreased splenic tissue acetylcholine levels while concomitantly increasing plasma acetylcholine (Figure 7), indicating that NC does modulate acetylcholine release, with the ultimate result of increased splenic clearance of this neurotransmitter, likely after its binding to downstream targets, and entry into peripheral circulation. Our previous studies have demonstrated that the spleen plays a central role in amplifying inflammatory responses and mediating myocardial IRI (2, 18), and that therapies to modulate the spleen to an anti-inflammatory phenotype attenuate myocardial IRI (11). Moreover, a cardioprotective role of the vago-splenic axis has been implicated in a preclinical study demonstrating infarct reduction *via* remote ischemic preconditioning in myocardial IRI, further establishing the role of both the spleen and vagus nerve in mediating the progression of infarction (13, 44). Taken together, these results suggest that the cardioprotective effect of NC may be attributable to activation of the vagal nerve, possibly *via* the CAP, to modulate splenic-derived inflammatory responses involved in post-ischemic myocardial reperfusion injury.

The extent of ischemic myocardial injury, defined by the duration from symptom onset to definitive treatment, determines post-ischemic reperfusion injury and therefore also final infarct size, which is the major predictor of clinical outcomes (2–5). Systemic therapeutic hypothermia attenuates ischemic myocardial injury and allows for longer ischemic time prior to reperfusion without consequent increase in infarct size (8–10). In this study we found that NC protects the heart not only from post-ischemic reperfusion injury but also from initial ischemic injury alone. During 40 min of LCA occlusion without reperfusion, NC attenuated ischemic myocardial infarct size by 50% compared to normothermic control as defined by TTC-blue staining (Figure 5) and significantly decreased CP levels of cTnT, cfDNA and HMGB1 (Figure 6). We have previously demonstrated that these two important DAMPs are released from ischemic myocardium during reperfusion and activate inflammatory pathways to induce reperfusion injury; both cfDNA and HMGB1 are vital to induce myocardial IRI (2, 18). The diminished release of cfDNA and HMGB1 from ischemic myocardium following NC mitigates the *de novo* inflammatory response within ischemic myocardium, as additionally reflected by the reduced CP levels of pro-inflammatory cytokines IFNα and IFNβ (Figure 6), and consequently is conducive to a reduced post-ischemic reperfusion injury as we have reported previously (18). Plasmacytoid dendritic cells are a potential target activated by cfDNA to produce IFNα and IFNβ and effect an injurious pro-inflammatory response (45–47). We plan to further evaluate the role of this innate immune cell subset in mediating myocardial IRI in future experiments.

Another mechanism through which NC-induced vagal activation may prove cardioprotective is through resulting bradycardia, given that NC demonstrated a significant decrease in HR. Bradycardia has long been thought to reduce cardiomyocyte oxygen consumption, and heart rate reduction therapies, including ivabradine or beta blockers such as metoprolol, given during the ischemic period of MI have been investigated with potential, but often equivocal, benefit (48–52). Such drugs may have significant benefit through alternate mechanisms independent of their induced bradycardia, such as with ivabradine (52). Within our study, late application of NC demonstrating decreased infarct size, albeit with less effect than when applied early, implicates a larger impact on the modulation of reperfusion injury. While our study underscores the impact of NC through inflammatory modulation, the effect of bradycardia cannot be excluded. The extent through which bradycardia plays an additional role warrants further investigation for neck cooling.

A limitation of this study is that the possibility of direct cooling of the heart contributing to NC's observed protective effect against ischemic injury and reperfusion injury cannot be excluded. We did find that NC resulted in moderate hypothermia in the pleural cavity (Figures 3A, 4B). Theoretically, NC may have directly lowered myocardial temperature either through local transfer from the neck subcutaneous tissues or return of cooled venous blood to the heart, and resultant reduced metabolic demands on the myocardium could, in part, account for the cardioprotective effect against ischemic and/or reperfusion injury. Studies with either large animals or more targeted cooling devices may help to differentiate these effects. Additionally, the role of the vagal cholinergic anti-inflammatory pathway in facilitating the cardioprotective effect of NC against ischemic myocardial injury and post-ischemic reperfusion injury remains to be defined.

In conclusion, NC without lowering core body temperature attenuated myocardial IRI to a similar degree to moderate systemic hypothermia. We demonstrated that NC reduces both ischemic and post-ischemic reperfusion injury *via* activation of the vagal cholinergic anti-inflammatory pathway. Clinically, NC may prolong the treatment window to achieve reperfusion without enlarging subsequent infarct size and would avoid the myriad negative side effects associated with systemic hypothermia. NC is a novel treatment that is straightforward to implement, readily accessible, and could be easily applied in the pre-hospital setting by non-medical personnel immediately after onset of acute MI to improve patient outcomes.

## Data Availability Statement

The original contributions presented in the study are included in the article/supplementary material, further inquiries can be directed to the corresponding author.

## Ethics Statement

The animal study was reviewed and approved by University of Virginia Animal Care and Use Committee.

## Author Contributions

AZ and ZY contributed to conception and design of the study. AZ, BY, DW, and ZY participated in acquisition of data *via* experiments. KM and ZY performed the statistical analysis. IK and ZY participated in funding acquisition. AZ wrote the first draft of the manuscript. AZ, KM, ZY, and RR wrote sections of the manuscript and performed critical revisions. ZY supervised all aspects of the study. All authors contributed to manuscript revision, read, and approved the submitted version.

## Funding

This work was supported in part by NIH R01HL130082, Commonwealth Health Research Board (CHRB) Grant Award #207-12-21, and University of Virginia George A. Beller, M.D. Research Award T32HL007849 (ZY) and T32HL007849-21A1 (IK).

## Conflict of Interest

The authors declare that the research was conducted in the absence of any commercial or financial relationships that could be construed as a potential conflict of interest.

## Publisher's Note

All claims expressed in this article are solely those of the authors and do not necessarily represent those of their affiliated organizations, or those of the publisher, the editors and the reviewers. Any product that may be evaluated in this article, or claim that may be made by its manufacturer, is not guaranteed or endorsed by the publisher.

## Abbreviations

CAP: cholinergic anti-inflammatory pathway
cfDNA: cell-free DNA
CP: cardiac perfusate
cTnT: cardiac troponin T
DAMP: Damage-associated molecular pattern
GEJ: Gastro-esophageal junction
HMGB1: High mobility group box protein 1
IFNα: Interferon alpha
IFNβ: Interferon beta
IRI: Ischemia-reperfusion injury
IS: Myocardial infarct size
LCA: Left coronary artery
LV: Left Ventricle
MI: Myocardial infarction
NC: Topical neck cooling
PCI: Percutaneous coronary intervention
PBS: phosphate buffered saline
TTC, 2, 3: 5-Triphenyltetrazolium chloride.
